# Approach to Pulmonary Arteriovenous Malformations: A Comprehensive Update

**DOI:** 10.3390/jcm9061927

**Published:** 2020-06-19

**Authors:** Shamaita Majumdar, Justin P. McWilliams

**Affiliations:** UCLA Department of Radiological Sciences, Los Angeles, CA 90095, USA; shamaitamajumdar@mednet.ucla.edu

**Keywords:** pulmonary arteriovenous malformations, hereditary hemorrhagic telangiectasia, transcatheter embolotherapy, screening, guidelines

## Abstract

Pulmonary arteriovenous malformations (PAVMs) are abnormal direct vascular communications between pulmonary arteries and veins which create high-flow right-to-left shunts. They are most frequently congenital, usually in the setting of hereditary hemorrhagic telangiectasia (HHT). PAVMs may be asymptomatic or present with a wide variety of clinical manifestations such as dyspnea, hypoxemia, or chest pain. Even when asymptomatic, presence of PAVMs increases patients’ risk of serious, potentially preventable complications including stroke or brain abscess. Transcatheter embolotherapy is considered the gold standard for treatment of PAVMs. Though previous guidelines have been published regarding the management of PAVMs, several aspects of PAVM screening and management remain debated among the experts, suggesting the need for thorough reexamination of the current literature. The authors of this review present an updated approach to the diagnostic workup and management of PAVMs, with an emphasis on areas of controversy, based on the latest literature and our institutional experience.

## 1. Introduction

Pulmonary arteriovenous malformations (PAVMs) are structurally abnormal, direct vascular communications between pulmonary arteries and veins, which bypass capillary beds to create low-resistance, high-flow continuous intrapulmonary right-to-left shunts [1,2,3].

The majority of PAVMs (70% or more) are associated with the autosomal dominant disorder hereditary hemorrhagic telangiectasia (HHT), also known as Osler-Weber-Rendu syndrome [2,4,5,6]. HHT mutations, which most commonly affect the *ENG* gene (HHT Type 1) or *ACVRL1* gene (HHT type 2), disrupt key regulators in angiogenesis, resulting in the development of congenital PAVMs and other vascular anomalies [1,7]. PAVMs affect about 50% of HHT patients overall [8], with a higher incidence and number of PAVMs in patients with *ENG* mutations [9]. Acquired causes of PAVMs account for approximately 20% of cases and include trauma, cardiothoracic surgery, hepatic cirrhosis, metastatic cancer, mitral stenosis, infection, amyloidosis, and chronic thromboembolic disease [2,3]. The wide variety of conditions associated with acquired PAVM suggests a common underlying developmental mechanism which triggers an angiogenic cascade within the pulmonary vasculature [3]. The remaining minority of PAVMs which cannot be classified as congenital or acquired are categorized as idiopathic.

PAVM angioarchitecture is classified as simple or complex based on the segmental pulmonary artery anatomy, which is important for planning endovascular interventions [5,10]. Simple PAVMs are supplied by a single segmental pulmonary artery. The single segmental pulmonary artery will often branch distally into one to three subsegmental branches all supplying the PAVM [5]. Complex PAVMs are supplied by two or more segmental pulmonary arteries. Diffuse PAVM is a rare subtype of complex PAVM characterized by involvement of an entire segment, or sometimes an entire lung, by tangles of malformed vasculature [3]. PAVMs often have a lower lobe predominance [11].

PAVMs are most frequently asymptomatic, but may be associated with a wide spectrum of clinical manifestations, and if left untreated, they can result in serious complications. Physiologic consequences correlate with the size of PAVM and degree of right-to-left shunting, which can limit oxygenation and natural filtration in the lung. Patients may present with varying degrees of dyspnea, cyanosis, clubbing, or chest pain [2,3]. Due to decreased filtration of vasoactive substances into the systemic circulation, migraines are a common neurologic manifestation of PAVM [1]. More serious complications include brain abscess, paradoxical embolism resulting in stroke or transient ischemic attack (TIA), and less frequently, hemoptysis or intrapulmonary hemorrhage [10,12]. HHT patients with PAVMs are several hundred-fold more likely to develop brain abscess compared to the general population [13,14]. The risk of brain abscess is significantly correlated with number of PAVMs, and larger feeding artery size is significantly correlated with risk of ischemic stroke [15]. Current indications for treatment of PAVM include any (solitary or multiple) PAVM with feeding artery diameter ≥2–3 mm, measurable increase in PAVM size, paradoxical emboli, symptomatic hypoxemia, or any of the other aforementioned serious complications [16].

Embolization is the standard of care for treatment of PAVMs, with surgery reserved for refractory cases which have repeatedly failed embolotherapy [17]. This review aims to present updated recommendations for the diagnostic workup and management of PAVMs, with an emphasis on screening protocol and aspects of care within particular subsets of patients, based on the most recent literature and the experiences at our institution, which has been an HHT Center of Excellence since 2010.

## 2. Methods

**Figure 1:**
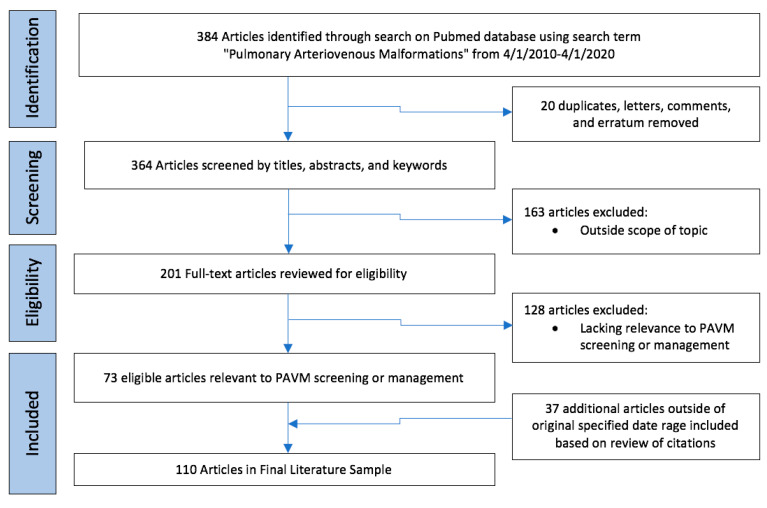
Flowchart for literature selection for narrative review.

A comprehensive narrative review of the last 10 years of literature relevant to pulmonary AVM diagnosis and management was undertaken using PubMed search term (pulmonary arteriovenous malformations) for date range 1 April 2010 to the date of review on 1 April 2020 (Figure 1). The literature search yielded 384 results. After excluding duplicative cohorts, letters, comments, and erratum, the remaining 364 articles were screened by titles, abstracts, and keywords. A total of 201 publications were identified as within the scope of the topic and the full texts were reviewed for eligibility. 73 articles were deemed relevant to PAVM screening and management. Citations of these 73 papers were examined for key articles which may have been published outside of the specified date range. Particular attention was given to articles relevant to areas of controversy, including PAVM screening, size criteria for PAVM treatment, management of PAVMs in pediatric and pregnant patients, treatment of persistent and diffuse PAVMs, management of PAVMs in the setting of pulmonary hypertension, and choice of embolic devices for PAVM embolization. The final literature sample included 110 articles relevant to PAVM screening and aspects of management, with a focus on areas on controversy.

## 3. Screening Protocol in Patient with Suspicion for PAVM

### 3.1. Transthoracic Contrast Echocardiography

The International Guidelines for Diagnosis and Management of HHT recommend that clinicians screen all patients with possible or confirmed HHT for PAVMs [18]. Transthoracic contrast echocardiography (TTCE) is the screening test of choice for PAVM, with a sensitivity of up to 98.6% and high rates of inter-observer agreement [19,20]. According to the 2011 International Guidelines, TTCE is considered positive if there is any detection of bubbles in the left atrium, and all positive screening tests should subsequently be confirmed with CT and recommended for antibiotic prophylaxis [18]. In patients with negative initial screening, repeat screening should be considered after pregnancy, within 5 years preceding planned pregnancy, and otherwise every 5–10 years. However, recent studies have brought into question whether confirmatory CT and antibiotic prophylaxis are necessary for mildly positive TTCE screening studies.

The TTCE shunt grading system is divided into a 0–3 scale, depending on the degree of left ventricular opacification after administration of a contrast agent. Grade 1 shunts demonstrate minimal opacification with less than 30 bubbles on any single frame. Grade 2 shunts correspond to moderate opacification with 30–100 bubbles. Grade 3 shunts have extensive opacification with >100 bubbles. A 2016 survey assessing current practices among 33 practitioners at HHT Centers of Excellence worldwide showed that for patients with Grade 1 screening echocardiograms, 41% recommend follow up with contrast enhanced CT, 22% recommend noncontrast CT, and 25% recommend repeat TTCE in 5–10 years, suggesting inconsistency among practices [21].

Velthuis et al. demonstrated a relationship between the grade of right-to-left shunt on TTCE and the prevalence of cerebral complications (ischemic stroke, TIA, brain abscess) in patients screened for HHT [22]. Out of 1038 patients, 530 had shunts detected on TTCE. Grade 1 shunts were not associated with an increased prevalence of cerebral manifestations, whereas grade 2 and 3 shunts were both independent predictors for prevalence of cerebral complication. 58 patients had shunting only with Valsalva maneuver, suggesting a diagnosis of patent foramen ovale (PFO) rather than pulmonary shunt, and none of these patients had any subsequent complication. Another study by Velthuis et al. prospectively investigated whether TTCE shunt grade could predict size of PAVMs on chest CT in 510 patients [23]. The positive predicted value for presence of PAVM on chest CT was 13.4% for grade 1 shunts, compared to 45.3% and 92.5% for grade 2 and 3 shunts, respectively. Moreover, none of the grade 1 shunts subsequently needed embolization, whereas 25.3% of grade 2 shunts and 77.4% of grade 3 shunts required endovascular closure of PAVM. Other studies have reported similar observations, for example that increased shunt grade predicts presence of PAVM on chest CT [24,25,26], and that patients with grade 1 shunts do not receive intervention, whereas those with grade 2 and 3 shunts often do [27,28,29,30].

These studies strongly suggest that grade 1 shunts are not associated with cerebral complications and do not predict presence of treatable PAVMs. As a result, we suggest that in patients with grade 1 shunts on screening TTCE, a conservative strategy withholding chest CT and antibiotic prophylaxis is justified and appropriate [31]. Repeat TTCE screening should be performed every 5 years to monitor for increased shunting. In patients with grade 2 shunts or higher on screening TTCE, diagnostic chest CT should be performed. Lastly, patients who demonstrate TTCE shunting only with Valsalva most likely have a diagnosis of PFO rather than PAVM, so the maneuver should be avoided during PAVM screening, and subsequent chest CT is not necessary.

### 3.2. Contrast Versus Noncontrast Thoracic CT

As mentioned previously, for patients undergoing confirmatory chest CT after screening TTCE, there is inconsistency among providers on whether to perform this study with or without contrast [21]. Currently, unenhanced multidetector chest CT with thin-cut 1–2 mm reconstructions is considered the gold standard for confirming PAVM [2,3,18]. Noncontrast CT is sufficient for identifying the highly characteristic feeding artery and draining vein, with a saccular or fistulous connection in between [10]. However, some institutions choose to perform CT with a modified pulmonary angiography protocol (CTPA) to better define the PAVM angioarchitecture and aid in treatment planning [2,3]. Furthermore, contrast can be a useful, though not absolutely necessary, tool for follow-up of embolized PAVMs, by assessing the presence or absence of contrast enhancement in the PAVM sac and/or draining vein. If contrast is used, care must be taken to avoid injection of air bubbles which could risk neurologic complication. 

Overall, we maintain that CT can be performed either with or without contrast for screening and diagnosis of PAVMs. The use of contrast can help screen for extrapulmonary vascular anomalies, better define PAVM angioarchitecture, and provide additional variables to assess treatment success on follow-up CT, but has the disadvantage of increased cost and possibility of air embolism, allergy or nephrotoxicity.

## 4. Management of PAVM by Sub-Populations

### 4.1. Small PAVMs

The 2011 HHT consensus guidelines recommended embolization of PAVMs with a feeding artery diameter 3 mm or greater, with the caveat that targeting PAVMs with a feeding artery as low as 2 mm may be appropriate in some cases [18]. Older literature commonly referenced the notion that cerebral ischemic events did not occur in patients having PAVMs with feeding artery diameter below 3 mm [32]. However, subsequent studies suggest that even small PAVMs can sometimes result in complications. One case in 2004 described embolization of a PAVM with feeding artery diameter of 1.8 mm in a patient with recurrent embolic strokes, with no further strokes post-treatment [33]. In 2004, Mager et al. reported long-term outcomes after embolization of 349 PAVMs in a cohort of 112 patients [34]. PAVMs targeted in this study had feeding artery diameter ≥3 mm or had caused bleeding or systemic complications. In 38 patients, smaller visualized PAVMs were initially left untreated; 16 of these 38 patients (42%) later required reintervention due to increasing shunt or complication. In 74 patients, all of the visible PAVMs including small lesions were treated at the initial procedure, and only 4% of patients in this group required reintervention. These data suggest that patients are much more likely to require reintervention when all visible PAVMs are not embolized at the time of initial procedure. Three of the five complications reported in the study were definitive sequelae of small untreated PAVMs, including one brain abscess and two TIAs. A 2006 study by Pollak et al. reported that nearly 20% of small PAVMs will grow over time, and that up to half of these may result in symptomatic events or complications [35]. A study in 2008 examining risk factors for stroke and brain abscess in HHT patients with PAVMs found that all patients who experienced stroke or abscess in spite of previous embolization had small untreated PAVMs with feeding artery diameter ≤2–3 mm, and embolization of all angiographically visible PAVMs was associated with significantly reduced ischemic stroke rate [36].

Overall, current evidence suggests that serious complications including stroke and brain abscess may occur in PAVMs with feeding artery diameter <3 mm. Given this risk, we feel it is reasonable to perform embolization for any PAVM with feeding artery diameter 2 mm or larger, and any symptomatic PAVM. Furthermore, several studies suggest that embolization of all angiographically visible PAVMs at the time of initial procedure will significantly reduce the likelihood of needing reintervention and risk of complication. Thus, if an embolization procedure is being performed, we recommend that all visible PAVMs (including those smaller than 2 mm) be occluded at the same session, if technically feasible.

### 4.2. Pediatric Patients

The 2011 International Guidelines recommend that PAVM screening be performed at the time of the initial clinical evaluation for HHT for both adults and children, with TTCE as the initial screening test and confirmatory CT performed for positive TTCE [18,37]. However, these guidelines pose some drawbacks for the pediatric population. As previously discussed, many studies have shown that TTCE is often positive in the absence of treatable PAVM, particularly for low grade shunts [23,24,25]. Since any positive TTCE study mandated subsequent CT, this potentially exposes the child to unnecessary or avoidable radiation. Furthermore, the placement of intravenous lines, which is necessary for TTCE, may be stressful for pediatric patients.

Mowers et al. completed a retrospective 14-year longitudinal study of PAVMs in 129 children with HHT [38]. The study utilized standard screening methods, with initial TTCE screening, followed by confirmatory CT for any positive TTCE shunt graded 2 or above. Negative patients were rescreened every 5 years, and all PAVMs with a supplying artery greater than 3 mm were embolized. 59% (76/129) of children screened positive on TTCE and were diagnosed with PAVM. Of those, 38 (50%) had small (<3 mm) PAVMs which were left untreated, and 38 (50%) had large PAVMs ≥ 3 mm which were embolized. 15 children had symptomatic PAVMs, all of which were large. All patients in the untreated cohort remained asymptomatic. Nine of the 38 treated patients had initial negative screening, but had PAVMs which grew larger at follow-up and were subsequently embolized. 21% (8/38) of the embolization group required repeat intervention, primarily due to persistence of the treated PAVM. No children in the study suffered treatment complications or adverse events in follow-up.

Hosman et al. completed an 18-year prospective study using a more conservative method of screening in 175 children with HHT [39]. In the conservative approach, screening was performed every 5 years using history and physical to detect dyspnea, cyanosis, and clubbing, pulse oximetry to detect hypoxemia, and chest radiography to screen for visible PAVM. Positive abnormalities were found in 50/175 (28%) children with HHT, and these 50 patients subsequently received diagnostic CT. 39 out of 50 patients who underwent CT were found to have PAVMs, suggesting highly efficient detection rate using this screening method. 33/39 (85%) children with PAVMs underwent embolization, 29 of them before the age of 18. 19/29 (66%) treated patients required multiple interventions due to persistence. 57 HHT patients who did not have PAVM detected through childhood screening received TTCE screening after age 18. In 6/57 (11%) of these patients, a PAVM was detected in adult screening and 2/57 (3.5%) were ultimately embolized. No children in the cohort suffered brain abscess, stroke, hemoptysis or hemothorax due to PAVM.

Based on these studies, we can conclude that both standard and conservative approaches are acceptable screening methods for PAVM in children. Standard screening with TTCE every 5 years, followed by noncontrast low-dose CT for grade 2 or higher shunts is safe and effective at detecting PAVM, but risks exposing a larger percentage of pediatric patients to radiation. Conservative screening with physical exam, pulse oximetry, and chest radiograph every 5 years is also safe and effective, with the added benefit of decreased likelihood of exposure to CT radiation. However, with the conservative algorithm, there is a small chance of missing a treatable AVM which might not be detected until adulthood. 

Regarding frequency of screening, the existing recommendations state that for patients with initial negative TTCE screen, screening should be repeated after puberty, and otherwise every 5–10 years. However, recent longitudinal studies suggest that PAVMs can grow during puberty and that a more frequent screening interval during childhood may be appropriate. Mowers et al. reported that, out of 31 children with HHT with negative initial screening who were followed for more than a year, nine of them (29%) went on to develop new PAVMS on subsequent TTCE screen, with a mean time to detection of 5.6 years [38]. Another study showed that, among 37 children with known PAVMs followed with interval CT scans, PAVM size seemed to grow approximately 10% per year and double in size every 5–6 years [40]. Since evidence suggests that PAVMs are expected to grow during childhood, increasing the risk of worsening symptoms or complications, we maintain that pediatric patients should be rescreened for PAVMs at 5-year intervals. 

In the pediatric population, there remains significant controversy regarding treatment approach. Current guidelines state that symptomatic children should always be treated, whereas treatment of asymptomatic children should be considered on a case-by-case basis. The prevalence and symptomatology of PAVMs in children is similar in frequency and distribution to adults, and serious complications from PAVMs do occur, particularly in PAVMs ≥ 3 mm [38,41]. One series followed 42 children with PAVMs treated with embolization (diameter ≥ 3 mm) for an average of 7 years [41]. Prior to clinical assessment or embolic treatment, several children within the study group had suffered serious complications related to PAVMs: 60% (25/42) of patients presented with cyanosis; hemoptysis had occurred in 7% (3/42) and neurologic complications had occurred in 19% (8/42). This study demonstrated significant improvement in oxygenation after embolization, particularly in patients with focal PAVMs, and only one post-treatment neurologic event, which occurred in a patient with diffuse PAVMs. Persistence at follow-up occurred in 15% of PAVMs, emphasizing the need for long-term follow-up post-treatment. Several other studies also suggest that PAVM-related complications in children tend to occur only with large PAVMs, and that small untreated PAVMs may not pose as significant of a threat as in adults [39,42,43].

Our own institutional practice is to screen infants and young children clinically using history, physical, and pulse oximetry, and to start TTCE screening at age 10–12, with repeat TTCE every 5 years thereafter. We prefer to treat symptomatic PAVMs and any PAVM ≥ 3 mm in pediatric patients, and to monitor smaller asymptomatic PAVMs until age 18 to reduce childhood radiation exposure. When embolization is required, we perform dense distal embolization of the PAVM to mitigate the high rates of persistence at follow-up.

### 4.3. PAVMs in Pregnancy

PAVMs have been observed to cause increased morbidity during pregnancy [44]. Physiologic changes of pregnancy result in increased blood volume and cardiac output, particularly in the second and third trimester, which may raise pressure within the PAVM. Moreover, high progesterone levels are thought to increase venous distensibility. These physiologic factors can promote enlargement and rupture of PAVMs during pregnancy. Numerous cases of PAVM-related complications during pregnancy have been reported in the literature, most frequently hemothorax [45,46,47,48,49,50,51]. However, despite acknowledgement of this greater risk during pregnancy, international consensus guidelines do not provide specific recommendations about treatment of PAVMs during pregnancy [18]. The British Thoracic Society guidelines advise clinicians to consider pregnancy a relative contraindication to elective embolization due to radiation and preterm labor risk, with the caveat that benefits may outweigh risks in setting of life-threatening hemoptysis [1]. They further contend that most PAVM pregnancies do well even in the setting of significant hypoxemia, but recommend management as a “high-risk” pregnancy, quoting a 1% risk of maternal death. However, our review of existing literature suggests this may be an underestimation, and that treatment of PAVM during pregnancy is both safe and warranted given the morbidity and mortality risk.

The risk of death with PAVM pregnancy quoted by the British Thoracic Society was determined from a 2008 review by Shovlin et al. [52]. This study reported 5 deaths out of 484 pregnancies in women with HHT, approximately 1%. However, out of 484 pregnancies, 23 cases (in 16 patients) were prospectively followed during the pregnancy, and 15 of the 16 patients in this prospective group had had their PAVMs embolized prior to pregnancy. Although no deaths occurred in the prospective group, inclusion of these pretreated cases is not a valid assessment of mortality risk for untreated PAVM. 239 of the 484 cases were previous pregnancies in women currently attending HHT clinic, constituting the retrospective study group. All patients in this subgroup had to be alive given they were present in clinic post-pregnancy, so their inclusion again is not valid to assess mortality. 222 pregnancies in the study were assessed in first-degree relatives with known HHT. In this group, 5 deaths occurred out of 222 cases, a rate of approximately 2.3%. The number of patients with PAVMs in this third study group was not known. Several may not have had PAVMs at all, and others may have had their PAVMs treated prior to pregnancy. Thus, estimates drawn from this study may underestimate the true mortality risk of untreated PAVM in pregnancy.

Earlier data does support the notion that untreated PAVMs are dangerous during pregnancy. A 1995 study by the same group reviewed maternal complications of 161 pregnancies in HHT women with and without PAVMs [53]. In 138 pregnancies without PAVM, no deaths occurred, and there was one ischemic stroke of unknown cause (0.7%). Of the 23 pregnancies with untreated PAVM, 8 (23%) resulted in nonfatal complications comprised of 6 pulmonary shunt increases and 2 ischemic strokes. 2 of the 23 PAVM cases resulted in fatal pulmonary hemorrhages, an 8.7% maternal death rate. 

A 2014 study surveyed women with HHT regarding complications during pregnancy, including 38 women with known PAVMs [54]. Eight women with PAVMs had been screened and treated prior to pregnancy, and no complications were noted in a total of 17 pregnancies in this group. Thirty women with PAVMs had not been treated pre-pregnancy; out of 64 pregnancies in this group, 11 complications were deemed to be PAVM-related, including 2 cases of hemoptysis, 5 hemothoraces (1 post-partum), 2 TIAs, and 1 post-partum myocardial infarction. While this survey-based study cannot be used to estimate mortality rate, the findings do suggest that untreated PAVM poses a high morbidity risk during pregnancy.

**Figure 2:**
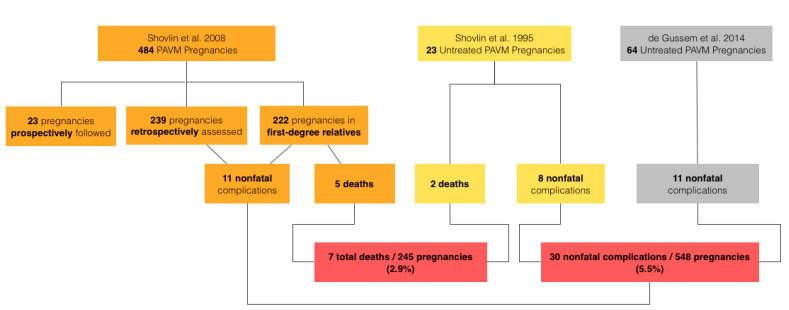
Flowchart representation of mortality and morbidity risk estimates for untreated pulmonary arteriovenous malformations in pregnancy.

The data from these three studies can be roughly combined to make conservative risk estimates for untreated pulmonary AVMs in pregnancy (Figure 2) [52,53,54]. A mortality estimate can be calculated based on the data from first-degree relatives in the 2008 survey (222 pregnancies) combined with the retrospective data from the 1995 study (23 pregnancies), which yields 7 deaths in 245 patients (2.9%). For non-fatal complications, a morbidity estimate can be calculated based on the retrospective data and the data from first-degree relatives in the 2008 survey (461 pregnancies), retrospective data from the 1995 study (23 pregnancies), and retrospective data from the 2014 survey (64 pregnancies), which yields 30 nonfatal complications in 548 pregnancies (5.5%). These risk estimates, although imperfect, do underscore the danger of untreated PAVMs in pregnancy. A recent review examining pregnancy in HHT concluded similarly that the current maternal mortality and morbidity risks quoted in the literature are likely underestimations, and that these cases should be considered high risk [51].

The British Thoracic Society describes pregnancy as a relative contraindication to elective embolization due to radiation exposure and risk of preterm labor [1]. However, studies have shown that complications from PAVM embolization are rare. One study examining outcomes of 205 PAVM embolization procedures in non-pregnant patients reported a ≤1% procedural complication rate, that being a single TIA [35]. Gershon et al. published a case series describing the embolization of 13 PAVMs in 7 pregnant patients, gestational age 16–36 weeks, reporting no complications [44]. Furthermore, there are no reports in the literature of PAVM embolization procedures causing preterm labor, adverse maternal outcome, or adverse fetal outcome. From the existing literature, the morbidity risk of PAVM embolization appears to be very low, and the mortality risk is essentially nonexistent. 

A second topic of frequent concern is radiation risk, but deterministic effects of radiation exposure in fetuses are only seen with high radiation doses [55]. The risks of radiation exposure decrease with gestational age. For example, at 8–15 weeks gestation, decreased intelligence quotient (IQ) is seen at 100 mGy and growth retardation at >250 mGy [55]. However, from 16 weeks gestation through term, decreased IQ is typically observed above 100 mGy and growth retardation is only observed at >1500 mGy [55]. The fetal radiation dose from PAVM embolization is estimated to be less than 1–2 mGy, well below all deterministic thresholds. Cancer risk is thought to increase by 0.01% for every 1 mGy of fetal radiation dose, meaning approximately 0.02% risk of cancer [55]. In other words, PAVM embolization has an estimated 1 in 5000 risk of cancer induction, and no deterministic effects, compared to a greater than 1 in 50 risk of maternal, and possibly fetal, death from untreated PAVM during pregnancy. From the existing evidence, we believe that the benefit of PAVM embolization during pregnancy greatly outweighs the risk. Regarding optimal timing of therapy, a review of 26 case reports of untreated PAVM complications in pregnancy found that 8% of complications occurred in the first trimester, 85% percent in the second or third trimester, and the remainder were unknown [44]. We feel that early in the second trimester may be the ideal time to treat pregnant mothers with PAVM, as this stage of fetal development has the least susceptibility to radiation and is generally a stable period with low risk of preterm labor.

A third area of concern in the context of pregnancy and angiography is the safety of contrast agents. In general, iodinated contrast media are considered safe for pregnant and lactating mothers, with the same risk factors for adverse reactions as the general population [56]. Although transplacental transfer of iodinated contrast has been observed, there is no evidence to suggest teratogenic effects in humans [56]. Nonionic iodinated contrast agents are preferred when contrast is needed in pregnant women, as they do not affect neonatal thyroid function [56].

Finally, it should be noted that PAVMs which were treated prior to pregnancy can have complications during pregnancy. In the aforementioned 2008 trial by Shovlin et al. 23 pregnancies in 16 patients with PAVMs were prospectively followed, and 15 of these patients had been embolized before pregnancy. Two nonfatal PAVM hemorrhages were observed in this group [52]. The previously discussed 2014 survey study reported no deaths and no complications in 17 pregnancies among 8 women treated prior to pregnancy. These numbers are too small to provide definitive risk estimates, but they should raise awareness that previously treated AVMs, especially those treated in the distant past and demonstrating systemic arterial reperfusion, can bleed during pregnancy. Any hemoptysis in a patient with treated pulmonary AVM should immediately raise suspicion for bronchial reperfusion with hemorrhage, and should prompt emergency room visit and definitive management.

**Figure 3:**
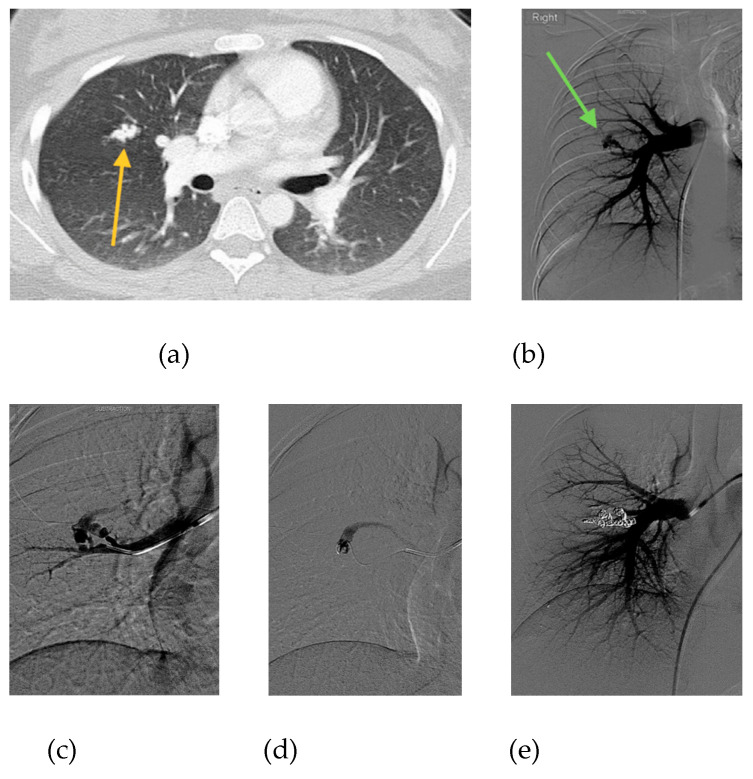
20-year-old female, who is 6 months pregnant presents with mild dyspnea. (**a**) Chest CT performed to rule out pulmonary embolism reveals complex PAVM with 3 mm feeding artery (orange arrow). (**b**) Initial pulmonary angiogram confirms PAVM in the right middle lobe (green arrow). (**c**) Angiogram shows selection of the supplying artery with a catheter. (**d**) Microcatheter placement into PAVM nidus. (**e**) Final angiogram shows complete occlusion of PAVM following embolization with 11 microcoils and 1 microvascular plug. Estimated fetal radiation dose was <5 mGy, scatter only with no direct irradiation. Remainder of pregnancy was uneventful and the patient delivered a healthy baby girl at 39 weeks gestational age.

In conclusion, evidence demonstrates that the risk of untreated PAVM in pregnancy is high, while the risk of PAVM embolization is vanishingly low. We believe that patients with untreated PAVMs should be treated prior to pregnancy whenever possible. Pregnant patients who have not been screened for PAVM should undergo screening with TTCE or low-dose noncontrast chest CT. If PAVMs are discovered during pregnancy, they should be treated. We have provided a case example of successful PAVM embolization during pregnancy from our institution (Figure 3). Patients who are symptomatic from their PAVMs should be treated immediately, regardless of gestational age. There is insufficient data to determine what PAVM size should be treated during pregnancy in an asymptomatic patient; our institution uses the same 3 mm feeding artery cut-off that we use for pediatric patients with asymptomatic PAVM, while patients with smaller PAVM are monitored closely and treated in the post-partum period. In patients with PAVMs treated during pregnancy, we recommend follow-up evaluation within 6 months postpartum, followed by a repeat screen every 3–5 years or prior to the next pregnancy. Patients with negative PAVM screening before or during pregnancy should continue to have standard repeat screening after pregnancy.

### 4.4. Persistent PAVMs

While embolization is the standard of care for PAVM treatment, up to 25% of initially successful cases experience persistence, meaning return of flow to the PAVM in follow-up [34,57,58]. Some providers believe that the presence of embolic material in persistent PAVMs can effectively filter clinically significant paradoxical emboli, rendering them less dangerous, while others theorize that persistent PAVMs may pose a higher risk owing to the potential for in-situ thrombus formation resulting from diminished flow [59,60]. The relative risk compared to native PAVMs is uncertain, but significant complications have been ascribed to persistent PAVMs, and re-treatment should be performed whenever feasible [57]. One commonly-used criterion for persistence is the failure of the draining vein or sac to regress by >70% on follow-up CT [57,61,62]. Persistence usually occurs in one of two patterns, recanalization or reperfusion. In recanalization, there is return of flow through previously placed embolic material supplying the PAVM. This is the most common pattern of persistence, seen in 88–91% of cases [57,63]. In reperfusion, flow reaches the PAVM by means of accessory arteries passing around the embolic material. Persistent PAVMs of either type have been shown to be difficult to treat, with variable success rates after repeat embolization ranging from 40% to 80% [35,57,63,64]. Studies report higher likelihood of retreatment success with recanalization compared to reperfusion [57].

A third, less common pattern of persistence is systemic-to-pulmonary reperfusion, usually arising from bronchial, internal mammary, or subclavian artery collaterals [57]. Although systemic-to-pulmonary reperfusion represents a left-to-left shunt and carries no risk of paradoxical embolization, the systemic pressure and fragile collateral arteries can lead to hemoptysis [35,58,63]. Asymptomatic patients can be counseled and monitored, while symptomatic patients should undergo systemic artery embolization or resection of the involved segment. Caution should be exercised during embolization of systemic-to-pulmonary collaterals, as multifocal strokes have been encountered from the use of particulate embolization [35].

The characteristics and treatment outcomes of persistent PAVMs described in the literature reveal several patterns which can be used to guide interventional management. Most complex PAVMs persist in a reperfusion rather than recanalization pattern, suggesting that at the time of initial embolization, thorough investigation of collateral branches and accessory feeder vessels to complex PAVMs should be performed, to reduce the risk of later reperfusion [65]. The most recent data reaffirms that persistent PAVMs are difficult to treat, but indicates that distal embolization beyond the existing embolic results in more durable occlusion [65]. This is consistent with similar findings from previous reports [57,63]. It is hypothesized that distal embolization may be more successful by allowing placement of embolic material directly into the PAVM sac or nidus, resulting in more durable occlusion regardless of angioarchitecture [65,66,67,68]. However, it should be noted that the distal embolization technique is not always technically feasible, since the previously deposited embolic material may prevent distal access [57,63,65]. One study reports a high success rate treating recanalized PAVMs using coils in conjunction with Amplatzer vascular plugs [61]. In either case, dense packing and complete stasis in the targeted AVM should be the desired endpoint [16].

Lastly, a recent study by Haddad et al. described a relationship between smoking and PAVM persistence at follow-up [69]. In 102 HHT patients with 373 treated PAVMs, five-year persistence-free survival rates in nonsmokers, smokers of 1–20 pack-years, and smokers of more than 20 pack-years were 12%, 22%, and 38% respectively [69]. The study demonstrated a dose-response and temporal relationship between smoking and PAVM persistence, likely related to effects on the vascular endothelium. While the total number of smokers in the study was relatively low and further studies are needed to confirm the findings, we recommend advising patients of these possible risks as further reason for smoking cessation. 

### 4.5. Diffuse PAVMs

Diffuse PAVMs are a rare subtype of complex PAVM, with a slight female predilection, in which one or more segments of the lung is diffusely involved by PAVMs [70,71]. Patients with this pattern of involvement can experience severe hypoxemia and are at far higher risk of serious complications including adverse neurologic events compared to those with focal PAVMs [70]. Due to the vast complexity of the malformation angioarchitecture, embolization in these patients is more technically challenging compared to standard embolization in focal or even multifocal PAVM cases.

Pierucci et al. has described the natural history and outcomes of 36 patients who underwent embolization for diffuse PAVMs [71]. Among the 10 patients with unilateral diffuse PAVM, 30% had experienced complications such as brain abscess or hemoptysis. Comparatively, of 26 patients with bilateral diffuse PAVM, 70% had had complications including abscess and stroke, suggesting a higher rate of adverse events with increasing lung involvement. Regarding procedural technique, the authors of study embolized all visible focal PAVMs with diameter >3 mm. In areas of diffuse PAVM, they utilized an approach called peripheral blood flow redistribution, treating only the most severely involved regions, using dense coil packing to perform peripheral-to-central occlusion of the target artery, thereby redistributing pulmonary blood flow to less involved portions of lung [71]. At a mean 8.5 year follow-up, oxygenation had significantly improved in both the unilateral cohort (from 87% to 95%) and bilateral cohort (79% to 85%). All nine deaths which occurred during the study period were in the bilateral cohort, 3 of which were PAVM-related complications (11%).

A second series reviewed 39 patients with diffuse or multifocal PAVM, including a subset of 22 patients with true diffuse PAVM, around 60% of whom had suffered neurologic complications [72]. This study used similar techniques, combining embolization of all large focal PAVMs and peripheral blood flow redistribution in areas of diffuse PAVM involvement. At mean 3.5 year follow-up, 80% of patients endorsed improvement in dyspnea symptoms. 10% of treated patients experienced ischemic or infectious complications due to reperfusion of embolized PAVMs or enlargement of untreated PAVMs.

Given the anatomic complexity and technical challenge of treating diffuse PAVM endovascularly, lung transplantation for diffuse PAVM has been described, typically in settings where PAVMs were not amenable to embolization or surgical resection [73,74,75]. The Registry of the International Society for Heart and Lung Transplantation reports a median survival around 8 years for lung transplant overall [76]. Most case studies on lung transplant for diffuse PAVM have only reported outcomes at short term follow-up, on the order of 1–3 years. A prospective study by Shovlin et al. reported long term outcomes of a small cohort of 6 patients with diffuse PAVM who were considered for lung transplantation [77]. The cohort was young (≤47 years), hypoxemic with baseline oxygen saturation less than 86%, and had all undergone maximal transcatheter embolization. One patient in the cohort received single lung transplant, but died within 4 weeks of surgery. The remaining 5 non-transplanted patients had a median 21 year survival, ranging from 16–27 years, considerably longer than the overall median survival reported for lung transplant. The marked longevity demonstrated by the non-transplanted cohort compared to the reported median survival with lung transplant is an important factor which patients and providers must consider; the option to pursue lung transplant for diffuse PAVM is a multifactorial decision which should give weight to survival outcomes, symptomatology, and quality of life in a case-by-case basis.

**Figure 4:**
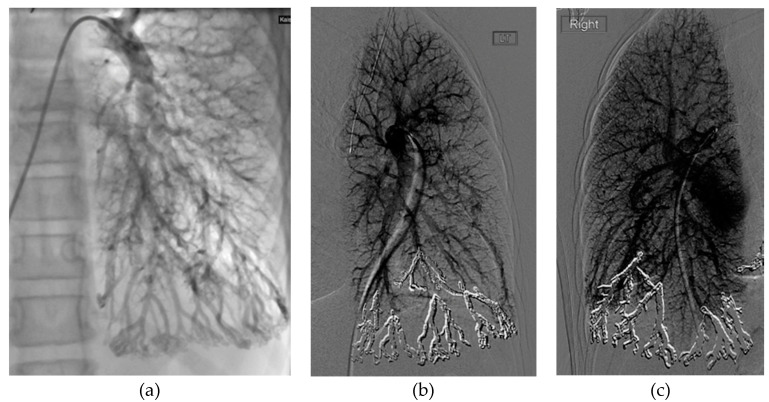
9-year-old female with genetically confirmed HHT, mild growth and developmental delay, presenting with dyspnea on exertion and chronic hypoxemia (baseline oxygen saturation of 60–80%) requiring supplemental oxygen. CT chest (not shown) showed diffuse PAVMs affecting all segments of both lungs. (**a**) Initial pulmonary angiogram of the left lung demonstrates diffuse PAVMs, most pronounced in the basal left lower lobe and lingula. (**b**) Final pulmonary angiogram of the left lung following peripheral-to-central embolization of the left lower lobe and lingula with implantation of 20 coils. (**c**) Final pulmonary angiogram of the contralateral right lung, performed 1 month after the left-sided embolization. Similar to the left lung, embolization was performed of the dominant PAVMs of the right lower lobe basilar segments. Despite the extensive embolization, the patient had no improvement in baseline oxygen saturation or functional status.

In patients with diffuse PAVMs, we believe that transcatheter embolotherapy should be first-line treatment. Embolization of all focal PAVMs ≥ 3 mm should be performed to reduce risk of stroke and brain abscess. Additionally, peripheral-to-central occlusion targeting areas of diffuse involvement can be considered, to achieve peripheral blood flow redistribution to less involved lung segments. This second approach can achieve modest improvements in dyspnea and hypoxemia in patients who have few lung segments involved. However, in patients with truly diffuse bilateral PAVM affecting all lung segments, transcatheter embolotherapy may not achieve meaningful improvements in hypoxemia, as there are no normal lung segments for redistribution (Figure 4). In these patients, embolization of only focal PAVMs ≥ 3 mm may be preferable, followed by expectant management. In all patients with diffuse PAVM, even after embolotherapy, adverse PAVM-related outcomes still occur with relatively high frequency; treated patients should be advised to seek prompt medical attention for symptoms suggestive of stroke, bleeding or brain abscess. 

### 4.6. Pulmonary Hypertension

Pulmonary hypertension (PH), defined as mean pulmonary arterial pressure (mPAP) ≥25 mmHg [78], is relatively common among HHT patients, with reported rates between 1.5–13% [10,79,80,81]. Screening for PH can be performed at the time of routine TTCE [79]. Many of these HHT patients have secondary PH, often as a result of high-output cardiac failure secondary to hepatic arteriovenous malformations (AVMs) [8,10,80,82]. A smaller proportion of patients, about 1%, have heritable or primary PH; this is usually seen with the *ACVRL1* mutation (HHT type 2) [83,84,85,86]. The coexistence of PAVMs and PH poses a clinical dilemma, as there is a paucity of data examining the evolution of PH following PAVM embolization. The presence of PAVMs may have a protective effect in the setting of severe PH, by providing a low resistance “pop-off valve” which could help decrease right ventricular afterload [10]. Treatment of PAVMs in these patients could hypothetically increase the pulmonary vascular resistance, worsening PH. Alternatively, the improved oxygenation and decrease in cardiac output following PAVM embolization could outweigh the potential increase in pulmonary vascular resistance, thereby mitigating an increase in pulmonary pressures [87,88,89].

**Figure 5:**
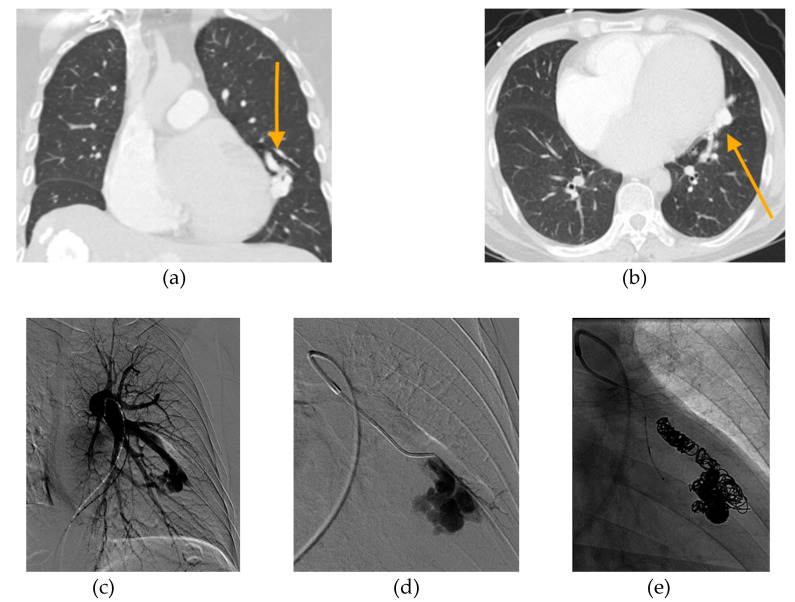
49-year-old male with history of nonischemic cardiomyopathy, severe mitral and triscuspid regurgitation, PAVM, and PH presented with acute hypoxemic respiratory failure. (**a**) Axial and (**b**) coronal CT angiogram images revealing a large PAVM in the lingula (orange arrows). (**c**) Initial pulmonary angiogram of the left lung demonstrates that a large proportion of pulmonary arterial flow passes through the PAVM, acting as a “pop-off” valve. (**d**) Selective angiogram shows selection of the feeding artery with filling of the complex PAVM sac. Prior to embolization, patient’s oxygen saturation on 4 L nasal cannula was 89%. Pre-embolization main pulmonary artery pressure (PAP) was 53/21 mmHg (mPAP 33 mmHg). (**e**) Final pulmonary angiogram shows occlusion of PAVM with combined coiling and deployment of an 8 mm Amplatzer plug in the arterial feeder. Following embolization, PAP increased 72/37 mmHg (mPAP 50 mmHg). Oxygen saturation on 4 L nasal cannula improved to 99%. The patient was weaned to room air and discharged in good condition. Two years later, he was admitted on multiple occasions for acute decompensated heart failure. At 3.5 years after embolization, he died from acute renal failure secondary to cardiorenal syndrome.

There are several conflicting reports in the existing literature. Cases have described fatal increases in pulmonary arterial pressure following PAVM embolization in the setting of severe baseline PH, defined by some as mPAP ≥ 40 mmHg [78,90]. Others report adverse outcomes of not treating, wherein worsening PH led to continued growth and eventual fatal rupture of untreated PAVM [91]. In a series of 43 patients, embolization of PAVMs did not generally lead to a significant increase in pulmonary artery pressure, in the setting of baseline mild-to-moderate PH [88]; notably, patients with baseline severe PH were excluded from the study. Within our own institution, we have experienced one case in which PH worsened following PAVM closure (Figure 5).

Before treating HHT patients with PH, one should seek to determine the etiology, and whether there is significant hepatic vascular involvement. Some reports suggest that occlusion of low-resistance PAVMs in the setting of high cardiac output could lead to worsening PH [58,90]. During PAVM embolization procedures in patients with PH, pre-embolization and post-embolization pressures can be obtained and compared. In cases where the effect of embolization is uncertain, temporary occlusion of the feeding artery can be performed with a balloon occlusion catheter while monitoring the cardiovascular response, to predict the risk of pulmonary hemodynamic changes prior to embolization [58]. There may also be a role for endothelin-receptor antagonists to mitigate worsening of PH following PAVM embolization in at-risk patients, though further study is needed [86].

We recommend that patients with coexisting PH and PAVM be considered for therapy on a case-by-case basis. Existing evidence suggests that embolotherapy of PAVMs is indicated in patients with mild-to-moderate PH. Though the data remains inconclusive, severe baseline PH (mPAP ≥ 40 mmHg) and larger PAVM size may result in increased likelihood of worsening PH after closure, and embolization in such cases should be carefully weighed. 

## 5. Embolic Devices

Coils are a well-established option for PAVM embolization, promoting luminal thrombosis through both reduction of vascular flow and intrinsic prothrombotic properties of the coil design. They are relatively easy to use and adapt exceptionally well to the shape of the vascular lumen. Regarding coil size, the diameter of the initial coil should not be smaller than the feeding artery diameter, as this increases the risk of paradoxical embolization [92]. Conversely, coils with too large of a diameter may lead to inadequate packing, or rarely complications such as vessel rupture [92]. For pushable coils, 20–30% coil oversizing relative to feeding vessel diameter is recommended [16,92]. With newer, longer detachable coil designs, coil size can be closely matched to the vessel diameter, as the longer coils provide more vessel wall contact to prevent migration. In general, long and soft coils can provide maximal packing density with low migration risk. However, currently published data do not demonstrate differences in safety, technical feasibility, or reperfusion rates at 1-year follow up based on type of coil utilized [93,94]. Some studies have reported high persistence rates for PAVMs treated with coils, up to 49% at 2-year follow-up [64]. A 2017 study by Stein et al. reported high persistence rates around 21% in small PAVMs ≤ 3 mm treated with coils alone [95]. Recent data suggests that all patients with embolization coils placed after 1984 can safely undergo 1.5 T magnetic resonance imaging (MRI) [96].

Amplatzer vascular plugs (AVPs) are dense expandable nitinol mesh vascular occlusion devices that reduce blood flow in a target vascular lumen to promote thrombosis [92]. It is recommended to oversize vascular plugs by 30–50% relative to the target vessel diameter at the occlusion site [92]. The vascular occlusion induced by AVPs is not instantaneous, and may take several minutes, particularly in high-flow settings [92]. Sometimes, the large amount of flow in the AVM can prevent occlusion or lead to later recanalization of the AVP; the addition of one or more coils proximal to the plug can help prevent this occurrence [61]. Compared to coils, AVPs appear to have a lower risk of device migration [3,97]. Vascular plugs may also have less metallic artifact compared to coils, which can be advantageous when evaluating treated PAVMs on follow-up imaging [3]. On the other hand, vascular plugs require rigid deployment systems with larger caliber sheaths and catheters, which can lead to technical difficulties, particularly limiting the ability to perform distal embolization [3]. One study evaluating efficacy of AVPs in PAVM treatment reported an 84% treatment success rate, defined as >70% sac size regression on follow-up CT [98]. A more recent study of 88 embolized simple PAVMs reported treatment success rates between 83.3–100% using various types of AVPs [99]. Other studies report low recanalization rates ranging between 5–7% [100,101]. One study found AVPs to be very effective in treatment of 24 large PAVMs with feeding artery diameter ≥8 mm, reporting no persistence, migration, or complications in follow-up [102]. Studies show that vascular plugs are less likely to recanalize compared to coils, and that vascular plug alone or in combination with coils might be a better primary option for PAVM embolization when technically feasible [16,103,104]. A 2012 study achieved high rates of technical success treating complex PAVMs by first performing venous sac embolization with detachable coils, followed by occlusion of the large feeding arteries using AVPs [105]. As with coils, AVPs placed after 1984 can be safely imaged using 1.5 T MRI [96]. 

Another device option for PAVM treatment is the Microvascular Plug (MVP) (Medtronic, Minneapolis, USA), a detachable plug consisting of a nitinol skeleton partially coated with polytetrafluoroethylene (PTFE) (Table 1). Advantages of MVPs in embolization of PAVMs include microcatheter-based deployment, resheathability, immediate occlusion even in the setting of intraprocedural anticoagulation, and less metal artifact compared to coils [106,107]. Furthermore, it is thought that the PTFE coating may help prevent delayed recanalization. Studies have shown the use of MVPs to be safe and technically successful in the treatment of PAVMs [106,108]. A 2015 series describing the use of MVPs to embolize 20 PAVMs in 7 patients demonstrated immediate cessation of flow through the feeding artery in 91% (21/23) of cases, with no device migration [106]. More recent studies have described similar findings using MVPs to treat PAVMs with feeding artery diameters >2 mm; they report technical success rates of 98–100% with immediate stasis of feeding vessels, and low persistence rates of 0–6%, considerably lower than rates seen with traditional coils [108,109,110]. A summary of the data presented in this section is provided in Table 1.

## 6. Follow-Up

Follow-up is important for patients with HHT to monitor for reperfusion of treated PAVM and growth of existing or previously microscopic PAVMs. As previously mentioned in the screening protocol discussion, patients with negative initial screening or suspected microscopic PAVMs (grade 1 shunts on initial screening TTCE) should have repeat TTCE screening every 5 years. 

In patients with initial negative screening CT, or a CT showing a very small PAVM not indicated for treatment, there is conflicting evidence on what constitutes an appropriate surveillance interval. Previous guidelines recommended 3–5 year CT follow-up. However, a 2019 study by Curnes et al. has reported a lack of growth over time for small untreated PAVMs in adults [111]. For each patient in the study, they compared 2 CT exams with the longest interval between them (mean 8.4 years, range 3.1–14.1 years) to assess growth, analyzing a total of 88 PAVMs in 21 patients. They found that untreated PAVMs grew slowly, if at all, and that any demonstrated growth was minimal and clinically inconsequential [111]. Similar findings were reported by Ryan et al. in a 2017 study investigating the natural history of small and microscopic untreated PAVMs in adults [112]. The findings from Curnes et al. and Ryan et al. challenge the guideline of 3–5 year CT follow-up for small untreated PAVMs, suggesting that this interval could be safety extended up to 5–10 years [111,112].

Another study assessed the diagnostic yield of rescreening adult HHT patients with initial negative screening CT [113]. They found that in 172 HHT patients, there is a low rate of newly detected PAVMs, approximately 0.7%/patient-year, most of which are small and not amenable to treatment. No treatable PAVMs were identified at the 5-year mark, and only 1 treatable PAVM was identified after 6 years, further supporting the notion that a longer screening interval of 5–10 years may be warranted [113]. In addition, a survey of providers at HHT Centers of Excellence worldwide has shown that around one fifth of providers already choose to obtain follow-up imaging in 10 years for patients who demonstrate PAVM stability on 2 CT scans in a 5-year period [21]. This is the regimen we follow at our institution.

The existing guidelines state that for patients who have undergone recent embolization of their PAVMs, follow-up CT should be performed within 6–12 months of treatment, then repeated every 3 years [18]. We feel that this interval can be increased in many patients, thereby reducing radiation exposure and reducing costs. At our institution, we recommend initial follow-up with CT within 6 months of embolization, followed by a repeat CT in 3–5 years based on likelihood of persistence, favoring shorter follow-up times for larger and more complex PAVMs. We use the common definition of successful PAVM treatment, that being more than 70% shrinkage of the draining vein or sac [57,61,62]. Either non-contrast or contrast-enhanced CT can be used.

One study has investigated whether graded TTCE can be used post-embolotherapy as a follow-up tool to predict the need for repeat treatment [114]. In 32 patients with prior PAVM embolization, graded TTCE was performed and the results were compared to their most recent chest CT. Two patients had PAVMs requiring repeat embolotherapy (feeding artery diameter ≥3 mm) due to untreated PAVM growth or treated PAVM persistence. All patients with negative TTCE had no visible PAVMs on CT. Both patients who did require repeat embolotherapy had grade 3 shunts on TTCE. The study suggests that post-embolotherapy TTCE can be used to predict the need for repeat embolotherapy and presence of treatable PAVM on CT. These results are promising and may provide an avenue for post-embolotherapy patients to avoid repeated radiation exposure.

Lastly, some authors suggest the use of time-resolved magnetic resonance imaging (MRI) for follow-up, particularly in patients treated with coils as there may be less induced metallic artifact with this modality [3,16,115,116]. A recent pilot study compared the use of ferumoxytol-enhanced MR angiography (MRA) to CT angiography (CTA) for PAVM detection [81]. The two modalities were comparable in detection rate for PAVMs > 2 mm, and ferumoxytol-enhanced MRA was able to detect several persistent PAVMs which were missed by CTA due to beam-hardening artifact from embolization coils. The data are preliminary, but both time-resolved MR and ferumoxytol-enhanced MR may prove to be feasible alternatives to CT for PAVM imaging, especially in the post-embolization setting, while avoiding the use of radiation and nephrotoxic contrast [117]. 

## 7. Conclusions

PAVMs are important to detect and challenging to treat. As the vast majority of PAVMs occur in the setting of HHT, particular attention should be given to screening and surveillance of PAVMs in this patient population. Detailed review of the current literature suggests that contemporary practices often deviate from previously published guidelines on PAVM management.

Grade 1 shunts on initial screening TTCE are not associated with cerebral complications and do not predict presence of treatable PAVMs, and therefore chest CT can be withheld. The same is true of shunts which are seen only with Valsalva maneuver. In these patients, TTCE screening can be repeated every 5 years.

Chest CT should be performed in patients with grade 2 shunts or higher on screening TTCE. CT can be performed sufficiently with or without contrast.

Serious complications including stroke and brain abscess often occur in PAVMs with feeding artery diameter ≥3 mm. These complications can also occur with smaller feeding artery diameters, though it is less common. It is recommended to treat any PAVM with feeding artery 2 mm or greater, and any symptomatic PAVM. Furthermore, embolization of all angiographically visible PAVMs at the time of initial procedure appears to significantly reduce the likelihood of reintervention and risk of ischemic stroke.

Both standard and conservative approaches are acceptable screening methods for PAVM in pediatric patients. Standard screening with TTCE, followed by CT for grade 2 or higher shunts, is safe and effective at detecting PAVM, but risks exposing a larger percentage of pediatric patients to radiation. Conservative screening with physical exam, pulse oximetry, and chest radiograph every 5 years is also safe, effective, and may reduce radiation exposure. However, with the conservative algorithm, there is a slightly higher risk of occasionally missing a treatable AVM which is not detected until adulthood.

PAVMs are expected to grow during childhood, potentially increasing associated risks and symptoms. Pediatric patients should be rescreened for PAVMs at 5-year intervals.

We recommend treatment of symptomatic AVMs and asymptomatic PAVMs ≥ 3 mm in pediatric patients, and monitoring of smaller asymptomatic PAVMs until age 18 to reduce childhood radiation exposure.

For untreated PAVM in pregnancy, both the morbidity and mortality exceed 1%, while the risk of PAVM embolization is much lower. Thus, pregnant patients with untreated PAVMs should be treated, prior to pregnancy if possible. If PAVMs are discovered during pregnancy, they should be treated. Symptomatic patients should be treated as needed, regardless of gestational age. Otherwise, early in the second trimester may be the ideal time to treat pregnant mothers with PAVM, as this stage of fetal development has the least susceptibility to radiation and is generally a stable period with low risk of preterm labor. Unscreened pregnant patients with HHT should undergo screening.

Persistent PAVMs are difficult to treat, with high rates of reperfusion or recanalization following repeat embolization. Embolization distal to the existing embolic results in a better rate of durable occlusion, especially when treating the recanalization pattern of persistence.

Patients with diffuse PAVMs are very high risk for adverse events. In patients with diffuse PAVM affecting only one segment or a few segments, the optimal treatment strategy combines embolization of focal PAVMs ≥ 3 mm and peripheral-to-central occlusion of the most severely affected segment(s) to achieve peripheral blood flow redistribution and improve hypoxemia. In patients with truly diffuse bilateral PAVM affecting all lung segments, attempts at blood flow redistribution are unlikely to achieve meaningful improvements in hypoxemia, though embolization of focal PAVMs ≥ 3 mm should be pursued to reduce risk of paradoxical embolization. Even after treatment, patients with diffuse PAVM remain high risk for complications.

Patients with coexisting PH and PAVM should be considered for therapy on a case-by-case basis. Treatment is typically indicated in patients with mild-to-moderate PH. Severe baseline PH should prompt careful consideration of risks versus benefits, due to the possibility of worsening PH after closure.

For patients with initial negative screening CT or small untreatable PAVM on CT, there is conflicting evidence regarding what constitutes an appropriate screening interval. The most recent studies demonstrate slow growth of small untreated PAVMs, challenging the recommendation to obtain follow-up CTs every 3–5 years, and suggesting that a longer screening interval of 5–10 years may be warranted for these patients.

For follow-up of treated PAVMs, we recommend initial follow-up with CT within 6 months of embolization. This should be followed by a repeat CT every 3–5 years based on perceived likelihood of persistence, favoring the 3 year follow-up interval for more complex PAVMs, and 5 years for all others.

Emerging data suggests that MR may have similar diagnostic accuracy as CT for follow-up of treated PAVMs, in particular for PAVMs with feeding artery diameter >2 mm. Preliminary data also suggests that serial graded TTCE has potential to be used as a post-embolotherapy follow-up tool, with high predictive accuracy for presence of treatable PAVMs on CT. These methods provide avenues to avoid repeated radiation exposure and nephrotoxic contrast.

## Figures and Tables

**Table 1:** Summary of literature on PAVM embolization outcomes of various embolic devices published in the last 10 years.

Study	Total # PAVMs Embolized	# Persistent PAVMs Embolized	Mean PAVM Feeding Artery Diameter (mm, ±SD) *	Embolic Devices	Technical Success **	Mean Follow-Up, Years	Persistence Rate at Follow-Up	Complications ^†^
Letourneau-Guillon 2010 [101]	35	0	5.0 (3.0–10.0)	AVP	97%	1.1	7%	Major: None Minor: Chest pain (4)
Trerotola 2010 [61]	37	0	Not reported (all ≥ 5 mm)	AVP + coils	100%	1.1	0%	Major: None Minor: Chest pain (7)
Tapping 2011 [100]	19	2	Not reported (3.0–12.0 mm)	AVP type I (8) AVP type II (11)	100%	2.3 (Type I) 1.5 (Type II)	5% (Type I) 0% (Type II)	Major: None Minor: Chest pain (1)
Hundt 2012 [105]	11	0	4.4 ± 1.4	AVP + coils	91%	Not specified	12.5%	Major: None Minor: Chest pain (4) Hemoptysis (1)
Kucukay 2014 [102]	24	0	11.5 ± 2.2	AVP	100%	3.0	0%	Major: None Minor: Chest pain (5)
Shimohira 2015 [64]	24	12	3.8 (1.4–5.2)	Coils	100%	2 (median)	49% (primary embolization) 100% (repeat embolization)	Not reported
Conrad 2015 [106]	20	0	3.5 (1.9–5.0)	MVP (19) MVP + coils (1)	100%	0.3	10%	Major: None Minor: Microemboli to toe (1)
Tau 2016 [104]	63	0	Not reported	Coils (37) AVP (21) AVP + coils (5)	100%	7.7	18.9% (coils) 0% (AVP) 0% (AVP + coils)	Major: None Minor: None
Stein 2017 [95]	141	0	2.4 ± 1.1	Coils	100%	1.6	21%	Major: None Minor: Chest pain (21) Groin infection (1) Hematoma (Not specified) Effusion (Not specified Flushing (Not specified)
Mahdjoub 2018 [108]	39	6	2.3 ± 0.7	MVP	98%	1.0	6%	Not reported
Andersen 2019 [103]	322	30	Not reported (all ≥ 2 mm)	Coils (213) AVP (89) Detachable balloon (13) AVP + coils (7)	100%	4.8	11.7% (Coils) 4.5% (AVP) 0% (Balloon) 14.3% (AVP + coils)	Not reported
Bailey 2019 [110]	119	0	3.3 ± 1.2	MVP	100%	0.9	0%	Major: None Minor: Chest pain (1)
Ratnani 2019 [109]	157	0	2.3 (1.0–5.9, MVP) 2.8 (1.0–7.6, Other)	MVP (92) Coils (24) AVP (35) AVP + coils (6)	100% (MVP) 100% (Coils) 97% (AVP) 100% (AVP + coils)	1.4 (MVP) 3.3 (Other)	2% (MVPs) 46.7% (Coils) 15% (AVP) 20% (AVP + coils)	Major: None Minor: Asymptomatic Pulmonary Infarcts (1)
Lee 2019 [98]	19	0	3.1 ± 0.7	AVP	100%	1.2	16%	Major: None Minor: Tachycardia (1) Chest pain (1)
Kennedy 2020 [94]	46	0	4.3 ± 1.5 (Nester) 4.4 ± 1.4 (Interlock)	Nester coils (26) Interlock coils (20)	100%	1.2	0% (Nester) 5.6% (Interlock)	Major: None Minor: Chest pain (5) Migraine (3) Minor hemoptysis (1)
Adachi 2020 [99]	88	0	4.1 ± 2.1	Coils (50) AVP (20) AVP + coils (18)	100%	3.2	22% (Coils) 10% (AVP) 39% (AVP + coils)	Major: None Minor: Not reported

* Range is provided when standard deviation was not specified. ** Technical success defined as complete angiographic occlusion of PAVM at end of procedure. ^†^ Value in parenthesis indicates the number of cases of a given complication. ^#^ Symbol defined as “Number of”.
